# NAD(P)H:Quinone Oxidoreductase 1 (*NQO1*) P187S Polymorphism and Prostate Cancer Risk in Caucasians

**DOI:** 10.3390/ijms130910959

**Published:** 2012-07-26

**Authors:** Christine G. Stoehr, Elke Nolte, Sven Wach, Wolf F. Wieland, Ferdinand Hofstaedter, Arndt Hartmann, Robert Stoehr

**Affiliations:** 1 Institute of Pathology, University Hospital Erlangen, Erlangen 91054, Germany; E-Mails: christine.stoehr@uk-erlangen.de (C.G.S.); arndt.hartmann@uk-erlangen.de (A.H.); 2 University Clinic of Urology, Friedrich-Alexander University Erlangen-Nürnberg, Erlangen 91054, Germany; E-Mails: elke.nolte@uk-erlangen.de (E.N.); sven.wach@uk-erlangen.de (S.W.); 3 Department of Urology, Caritas St. Josef Medical Center, University of Regensburg, Regensburg 93053, Germany; E-Mail: wwieland@caritasstjosef.de; 4 Institute of Pathology, University of Regensburg, Regensburg 93053, Germany; E-Mail: ferdinand.hofstaedter@klink.uni-regensburg.de

**Keywords:** prostate cancer, NQO1, case-control study, restriction fragment length polymorphism analysis

## Abstract

NAD(P)H:quinone oxidoreductase 1 (NQO1) catalyses the reduction of quinoid compounds to hydroquinones, preventing the generation of free radicals and reactive oxygen. A “C” to “T” transversion at position 609 of *NQO1*, leading to a nonsynonymous amino acid change (Pro187Ser, P187S), results in an altered enzyme activity. No NQO1 protein activity was detected in *NQO1* *^609^TT* genotype, and low to intermediate activity was detected in *NQO1* *^609^CT* genotype compared with *^609^CC* genotype. Thus, this polymorphism may result in altered cancer predisposition. For prostate cancer, only sparse data are available. We therefore analyzed the distribution of the *NQO1 P187S* SNP (single nucleotide polymorphism) in prostate cancer patients and a healthy control group. Allelic variants were determined using RFLP analysis. Overall, 232 patients without any malignancy and 119 consecutive prostate cancer patients were investigated. The genotype distribution in our cohorts followed the Hardy–Weinberg equilibrium in cases and controls. The distribution of the *NQO1* codon 187 SNP did not differ significantly between prostate cancer patients and the control group (*p* = 0.242). There was also no association between the allelic variants and stage or Gleason score of the tumors. The *NQO1 P187S* SNP was not significantly associated with an increased prostate cancer risk in our cohorts. The SNP has also no influence on histopathological characteristics of the tumors. A combined analysis of all available data from published European studies also showed no significant differences in the genotype distribution between controls and prostate cancer patients. Our data suggest a minor role of the *NQO1* nucleotide 609 polymorphism in prostate carcinogenesis.

## 1. Introduction

Oxidative stress represents a cellular situation where the production of reactive oxygen species (ROS) outruns the cell’s capacity to metabolize ROS, resulting in its accumulation and an increased possibility of DNA damage [1]. Therefore, each cell uses an antioxidant defense system to prevent ROS overproduction.

Oxidative stress and ROS signaling may play important roles in the development of several malignancies, including prostate cancer (PCa), and there is striking evidence for a potential role of increased ROS generation and its potential impact on etiology and progression of PCa [2]. An important factor of the cellular antioxidant defense system is the NAD(P)H:quinone oxidoreductase 1 (NQO1). NQO1 represents a widely distributed FAD-dependent flavoprotein with multiple protective functions. Among others, NQO1 catalyzes the reduction of quinones, quinoneimines, nitroaromatics and azo dyes. This reduction minimizes the opportunities for generating reactive oxygen intermediates by redox cycling in the cell, underlining the significant impact of NQO1 on cellular protection [3].

Within the *NQO1* gene located on chromosome 16q22.1, a prominent single nucleotide polymorphism (SNP) can be found. A “C” to “T” change at position 609 (C^609^T) of the *NQO1* cDNA results in a non-synonymous amino acid change from proline to serine at position 187 (P187S). This amino acid substitution leads to an extremely unstable NQO1 protein which is rapidly ubiquitinated and degraded by the proteasome [4]. Therefore, individuals with the *^609^TT* genotype lack both NQO1 protein and activity [5]. The *C^609^T* SNP is associated with an increased risk for several malignancies, e.g., colorectal cancer, breast cancer, lung and bladder cancer [6–8]. For PCa, only sparse and conflicting data are available. Four studies (three case-control studies on Caucasian cohorts, 1 case-control study on a Japanese cohort) reported no influence of the *NQO1 C^609^T* SNP on PCa risk [9–12]. In contrast, Steinbrecher and colleagues reported a significant, reduced PCa risk for subjects with the *^609^CC* genotype compared to *^609^CT* and *^609^TT* carriers in a German case-control study [13]. Unfortunately, the allele distribution of the control cohort in this study did not reach the Hardy–Weinberg equilibrium; therefore, the results of this study should be interpreted carefully. To advance this discussion we performed a case-control study on 119 PCa patients and 232 male controls using restriction fragment length polymorphism analysis (RFLP). In addition, we combined our results with the data from all published European studies and re-analyzed these data in a meta-analysis.

## 2. Results and Discussion

### 2.1. Results from Method Validation and Cohort Testing

**Figure 1:**
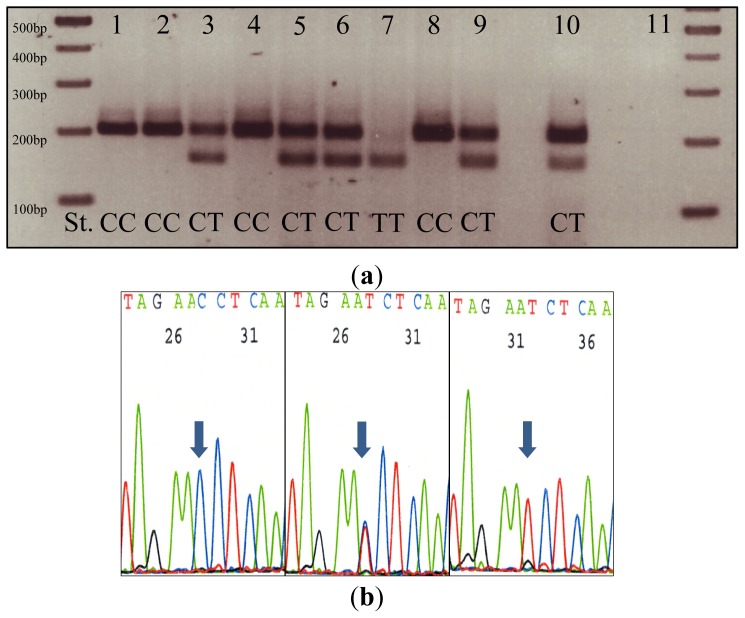
(**a**) Representative example for RFLP analysis. St.: size standard; 1–9: DNA from prostate cancer patients; 10: positive control (DNA from bladder cancer cell line RT4); 11: negative control (H_2_O); (**b**) Results from sequencing analysis of samples 4, 5 and 7 from (**a**). Both methods showed concordant results.

The verification of RFLP analysis by sequencing showed 100% concordance between both methods. All results derived from RFLP analysis could be confirmed by sequencing. Representative examples of genotyping are shown in Figure 1a; representative sequencing results are shown in Figure 1b.

**Figure 2:**
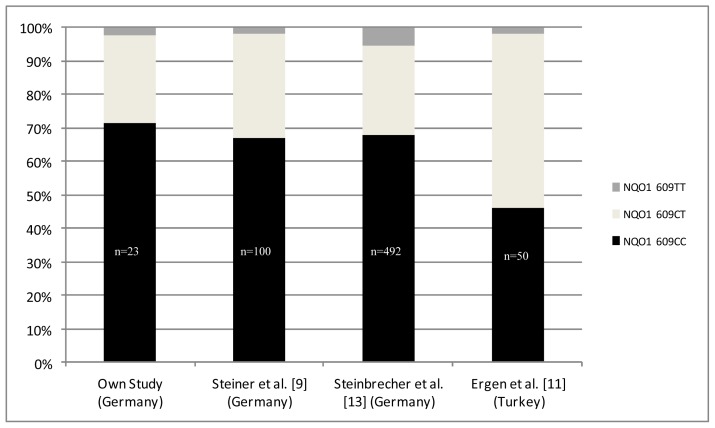
Distribution of the *NQO1 C^609^T* polymorphism published European male control cohorts.

The genotype distribution in our cohorts followed the Hardy–Weinberg equilibrium in cases (*p* = 0.578) and controls (*p* = 0.803). To ensure that our control group is representative for Caucasians, we compared the SNP distribution in our controls with published Caucasian control cohorts (Figure 2). Similar genotype distribution among the published cohorts from Germany underlined data integrity of our study. Allele frequencies from the Turkish study varied remarkably, which was presumably due to a small study cohort (*n* = 50 [11]).

### 2.2. Results from Our Single Center Analysis

**Figure 3:**
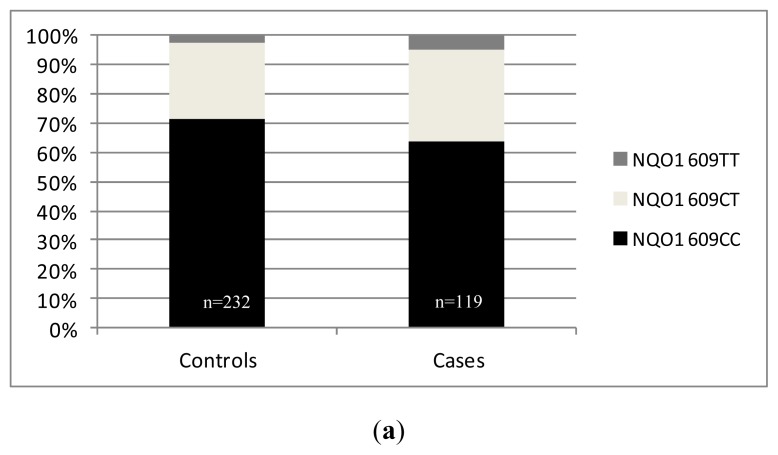

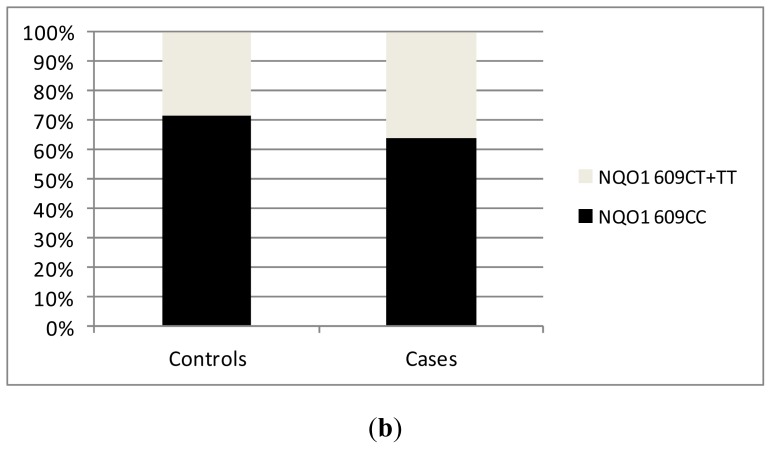
(**a**) Distribution of the *NQO1 C^609^T* polymorphism in our cohorts (*p* = 0.242); (**b**) Distribution of the putative risk allele *NQO1* *^609^T* in our study population (*p* = 0.146).

Genotype distribution did not differ significantly between cases (^609^CC: 63.9%, ^609^CT: 31.1%, ^609^TT: 5.0%) and controls (^609^CC: 71.6%, ^609^CT: 25.9%, ^609^TT: 2.5%) (*p* = 0.242) in our cohorts (Figure 3a). Referring to the putative risk allele *^609^T* (S187), there was also no significant difference in genotype distribution between the analyzed cohorts (cases: *^609^CC*: 63.9%; ^609^CT + ^609^TT: 38.1%; controls: *^609^CC*: 71.6%, ^609^CT + ^609^TT: 28.4%; *p* = 0.146; OR: 1.423; 95%CI: 0.889–2.278) (Figure 3b). In addition, there was also no correlation between SNP distribution and histopathological characteristics of the tumors.

### 2.3. Results from Combined Data of Published European Studies

**Figure 4:**
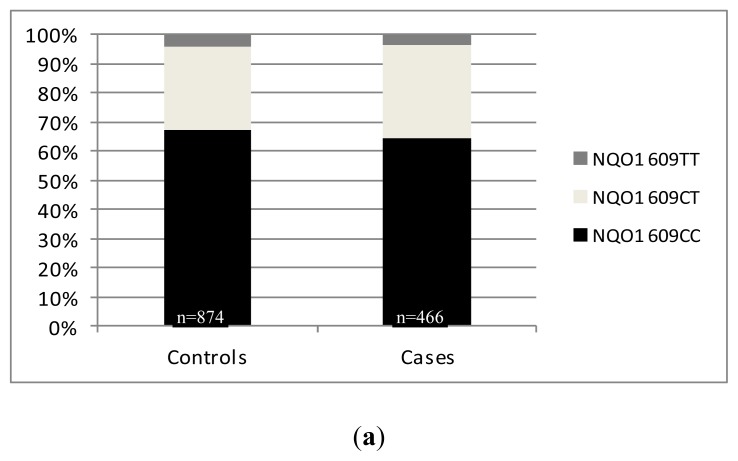

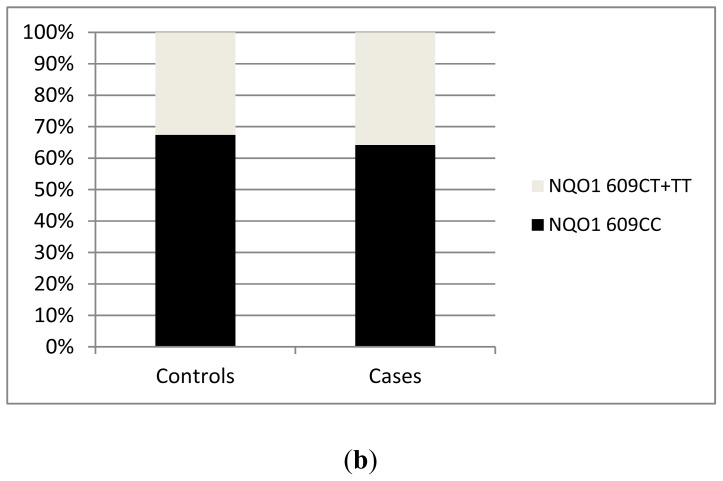
(**a**) Distribution of the *NQO1 C^609^T* polymorphism and the putative risk allele *NQO1* *^609^T*; (**b**) in healthy controls and PCa patients from all published European studies.

After the analysis of our cohorts, we wanted to increase the significance of the data by combining our results with available data from all published European studies on the influence of the *NQO1 C^609^T* SNP on PCa risk. This combination allowed the analyses of data from 874 control subjects and 466 prostate cancer patients. The genotype distribution in both cohorts followed the Hardy–Weinberg equilibrium in cases (*p* = 1.000) and controls (*p* = 0.213). The genotype distributions were as follows: cases: *^609^CC*: 64.2%, ^609^CT: 32.0%, *^609^TT*: 3.8%; controls: *^609^CC*: 67.4%, *^609^CT*: 28.6%, *^609^TT*: 4.0%; (Figure 4a). These differences in allele frequencies did not reach statistical significance (*p* = 0.438). Referring to the putative risk allele *^609^T* (S187), there was also no significant difference in genotype distribution between the combined analyzed cohorts (cases: *^609^CC*: 64.2%; *^609^CT* + ^609^TT: 35.8%; controls: *^609^CC*: 67.4%, *^609^CT* + *^609^TT*: 32.6%; *p* = 0.249; OR: 1.154; 95%CI: 0.911–1.462) (Figure 4b).

## 3. Discussion

There is strong evidence for the relationship between SNPs in genes coding for members of the antioxidant defense system and PCa. Among others, allelic variants of the superoxide dismutase and the catalase genes were described as associated with an increased risk for PCa development [14]. For the *NQO1 C^609^T* SNP, there is still an ongoing discussion about its impact on PCa risk. The presented data showed no involvement of this SNP in PCa risk and strengthened results from previous studies performed on smaller cohorts or on cohorts whose allele frequencies did not reach the Hardy–Weinberg equilibrium. In addition, the presented meta-analysis displayed the largest case-control study on the *NQO1 C^609^T* SNP in Caucasian PCa patients reported so far. Therefore, it might be assumed that the presented study contributed substantially to the discussion about the role of this *NQO1* SNP in PCa risk.

Nevertheless, the NQO1 protein might play an important role in PCa etiology. NQO1 is constitutively expressed in normal prostate tissue and is therefore capable of activating pro-carcinogens to reactive DNA-damaging metabolites [15]. High levels of reactive species increase oxidative stress in the cell, resulting in chronic inflammation, damage of nucleic acids and proteins, and deregulation of androgen receptor signaling [16]. All these effects are known to promote PCa development and progression. Unfortunately, first promising results in PCa chemoprevention with antioxidants in the preclinical settings could not be validated in clinical translation [16].

In PCa, NQO1 is overexpressed, compared to adjacent normal tissue, in approximately 60% of the analyzed cases, although the chromosomal region of the *NQO1* gene was described as frequently affected by deletions [17,18]. This overexpression might be utilized for beta-lapachone-induced apoptosis and cell toxicity. Beta-lapachone is a naturally occurring *o*-naphthoquinone showing anti-tumor properties and induces apoptosis in a variety of cells [19]. PCa cells were described as being killed by beta-lapachone via NQO1 metabolic bioactivation, resulting in e.g., a massive production of ROS and poly(ADP-ribose) polymerase-1 (PARP-1) hyperactivation. PARP-1 hyperactivation initiates programmed necrosis and was shown in massive, ROS-induced injury [20]. Moreover, the clinical utilization of beta-lapachone as a possible radiosensitizer in NQO1-overexpressing PCa to prey PARP-1 hyperactivation was suggested after promising *in vitro* studies [17]. In this context, the *NQO1 C^609^T* SNP might play an important role. In tumors overexpressing the non-active serine-variant of *NQO1*, this sensitizing effect should not be expected. This gives a possible rationale for the determination of the *NQO1* genotype at position 609 in PCa, because without knowledge of the allele status, this therapeutic option might remain ineffective. But, to date, this rationale remains speculative and convincing *in vitro* or *in vivo* data are missing.

Most recently, crucial, but ambiguous roles of NQO1 in PCa cells were demonstrated. Stable expression knockdown of *NQO1* resulted in both increased cell proliferation and sensitivity to oxidant treatment. Moreover, deregulated expression of more than 1600 genes after *NQO1* knockdown was determined. Interestingly, expression of genes of the pro-inflammatory network (e.g., *IL-8*, *IL1R2*) increased, whereas expression of members of the melanoma tumor specific antigen gene family decreased. Unfortunately, the mechanism by which *NQO1* might regulate this differential gene expression is still unclear [21].

In summary, the presented study showed no influence of the *NQO1 C^609^T* polymorphism on PCa risk in Caucasians. Nevertheless, data from actual literature indicate important functional roles of NQO1 in both prostate carcinogenesis and possibly therapy.

## 4. Experimental Section

### 4.1. Samples

Overall, 119 unselected PCa patients, who all underwent radical prostatectomy, were included in our study. Formalin-fixed and paraffin-embedded tissue samples from these patients were available from the prostatectomy specimens. For comparison, 232 blood or tissue samples from a male control group of patients without any malignancy were investigated.

All tumors were diagnosed according to the 2004 WHO classification of prostate tumors [22] and staged according to the TNM system [23]. Clinicopathological characteristics of the study participants are shown in Table 1. Internal Review Board approval was obtained for the study.

### 4.2. Tissue Microdissection and DNA Isolation

DNA was extracted from manually microdissected, histopathological inconspicuous tissue or peripheral blood using the High Pure PCR Template Preparation Kit (Roche GmbH, Mannheim, Germany) according to manufacturer’s instructions.

### 4.3. *NQO1 P187S* SNP Analysis

SNP analysis was carried out by restriction fragment length polymorphism analysis (RFLP) of the polymorphic region in exon 6 which contains a *Hin*fI recognition site (5′-GANTC-3′) in presence of the ^609^T-allele (S187). The presence of the *S187* allele resulted in a digest of the PCR product into three fragments (230 bp → 151 bp + 44 bp + 35 bp), whereas PCR products containing the ^609^C-allele (P72) resulted in two fragments (230 bp → 195 bp + 35 bp).

### 4.4. Amplification of *NQO1* Variants and RFLP Analysis

SNP region was amplified by PCR using primers (sense: 5′-TCC TCA GAG TGG CAT TCT GC-3′; antisense: 5′-TCT CCT CAT CCT GTA CCT CT-3′) obtained from Metabion (Martinsried, Germany) in a total volume of 25 μL containing 100 ng DNA, 0.2 mM dNTP (Promega), 0.18 μM primers and 0.0025 U/μL GoTaq (Promega, Mannheim, Germany). The thermal cycling conditions were as follows: initial denaturation for 3 min at 94 °C, 35 cycles of denaturation at 94 °C for 1 min, annealing at 58 °C for 45 s, elongation at 72 °C for 45 s and final primer extension at 72 °C for 10 min. Gradient

PCR was used for optimization of cycling conditions. PCR products were incubated for 4 hours with 10 U *Hin*fI (New England Biolabs, Frankfurt/Main, Germany) at 37 °C in a total volume of 30 μL to ensure complete digestion. Restriction fragments were separated by electrophoresis using 3.5% agarose gels and visualized under ultraviolet light by using 0.05% ethidium bromide.

### 4.5. Sequencing Analysis

Nine randomly selected cases were sequenced to verify the RFLP results. After amplification, PCR-products were purified using the Qiagene Dye Ex 2.0 TM Spin Kit according to manufacturer’s conditions. Sequence analysis was performed with primers mentioned above using Applied Biosystems Big Dye Terminator Cycle Sequencing Kit (version 1.1; Applied Biosystems, Austin, TX, USA, 2012) and an Applied Biosystems Genetic Analyzer (version ABI 3500 CE-IVD; Life Technologies Corporation, Carlsbad, CA, USA, 2011).

### 4.6. Statistical Analysis

To test if the genotype distribution followed Hardy–Weinberg equilibrium, a public software from the Institute of Human Genetics, Helmholtz Center Munich, Germany was used [24]. Chi square test (two-sided exact) was used to evaluate case-control differences in the distribution of genotypes and to analyze associations between genotypes and age or histopathological characteristics. To determine the distribution of the risk allele *versus* non-risk-allele Fisher’s exact test (two-sided) was used. *p*-values < 0.05 were interpreted as statistically significant.

## 5. Conclusions

The *NQO1 C^609^T* polymorphism seems not to play an important role in the risk for PCa development in Caucasians. Possible effects on disease aggressiveness, progression and therapy are yet to be determined in more detail.

## Figures and Tables

**Table 1:** Characteristics of study participants.

	Cases		Controls	
Number	*n* = 119		*n* = 232	
Age (years)	Median: 66	Range: 46–74	Median: 69.5	Range: 25–94
	Mean: 64.3 (± 6.4)		Mean: 67.9 (± 10.2)	
Stage	Organ-confined	*n* = 59		
	Non-Organ-confined	*n* = 55		
	No data available	*n* = 5		
Gleason Score	Median: 7	Range: 3–10		
Gleason Sum	3–4	*n* = 3		
	5–7	*n* = 80		
	8–10	*n* = 28		
	No data available	*n* = 8		
